# Endometriosis and infertility: the hidden link between endometritis, hormonal imbalances and immune dysfunctions preventing implantation!

**DOI:** 10.5935/1518-0557.20230015

**Published:** 2023

**Authors:** Patrick JD Bouic

**Affiliations:** 1 Synexa Life Science, Cape Town, South Africa

**Keywords:** endometritis, immune dysfunction, inflammation, oestrogen-metabolism disturbance

## Abstract

Endometriosis is a chronic inflammatory condition affecting up to 10% of women of reproductive age and this, depending on its severity, very often leads to infertility. New research has shed light on the role of underlying endometritis due to the presence of inflammatory, non-oestrogen metabolising microbiome at the mucosal interface and this in turn leads to the activation of aggressive, non-tolerant immune cells in the endometrium. These immune cells require the presence of tolerance-inducing commensals such as *Lactobacilli* so as to allow the implantation of the fertilised egg. New therapies should be holistic and address both the dysbiosis as well as immune abnormalities. Routine immune monitoring of the immune cells derived from the endometrium and/or microbial profiling should recommended to better predict assisted reproduction outcomes in these couples.

## INTRODUCTION

Chronic endometriosis is a devastating condition affecting up to 10% of women of reproductive ages (up to 196 million women globally) and it is characterised by extensive growth of endometrial tissue and stoma outside of the uterus. The presenting symptoms include unexplained infertility, pelvic pain, excessive bleeding and pain upon urination or intercourse or even bowel movements. The current medical management of such cases include repeated surgery to remove the ectopic endometrial tissue and/or hormonal therapy but these approaches seem ineffective in preventing recurrences.

The current knowledge of the aetiology of endometriosis is limited but the principal theory is that endometrial tissue implants at ectopic sites far from the uterus but normally the immune cells at such sites are responsible for the clearance of such lesions. In 10% of women, it appears that such immune clearance is ineffective leading to the establishment and proliferation of the tissue leading to the lesions. Chronic inflammation in the peritoneal cavity causes the spread of the lesions.

What then is the link between hormone metabolism, the local microbiome and the pro-inflammatory profile displayed by most women suffering from this condition?

## THE MICROBIOTA AND HORMONE METABOLISM

Numerous recent studies have shown chronic endometritis as a common feature of women presenting with endometriosis. Most women present with altered microbiota and the first reports date back to 2014 when these authors showed significant differences in the uterine ad cervical microbiome communities between women with endometriosis and those without (Takebayashi *et al*., 2014). Shan *et al.* (2021) subsequently observed lower alpha diversity of gut microbiota and a higher Firmicutes-to-Bacteroidetes ratio in women with stage 3/4 endometriosis (n=12) than healthy controls. Such a scenario promotes a pro-inflammatory response and a dysregulated immune profile in the affected women eventually leady to infertility and implantation failures. Endometriotic microbiota of affected women has been consistently associated with diminished *Lactobacillus* dominance and the overgrowth of inflammatory species such as *Firmicutes*, elevated *Gardnerella, Streptococcus, Escherichia, Shigella,* and *Ureoplasma* in their cervix and elevated *Shigella/Escherichia* in their stool (Singh & Sethi, 2022).

Lessons learnt form studies conducted in Irritable Bowel Syndrome (IBS) and the obvious microbiome dysregulation can be applied directly to the microbiological profile seen in endometriosis and chronic endometritis. The role of the microbiota in possibly driving local and systemic inflammation and the relationship to the pathophysiology of multiple gynaecologic conditions remains under investigation (Jiang *et al*., 2021). The pathogenesis of endometriosis and comorbidities is likely related to the interplay of multiple environmental factors, genetic factors, inflammation, immune dysregulation, hormonal imbalance and possibly the microbiome (Chadchan *et al*., 2023). Chronic activation of the immune response can result in pelvic inflammation and severe endometriosis can result in pelvic adhesive disease.

The microbiome relates to the collection of genomes of microorganisms in a particular environment or mucosal site, such as the GI, cervicovaginal and pulmonary mucosa. The gut microbiome plays a key role in physiological processes, including nutrient absorption, maintaining the integrity of the GI lining, regulation of immune and endocrine systems, and protection against pathogenic insults. An ‘optimal microbiome’ can maintain the well-being and homeostasis of the individual and this microbiota composition confers health benefits at mucosal sites. In addition, the gut microbiota can influence host health through mediating changes in the metabolome. The metabolome is composed of all the metabolites present in a particular environment. A diverse collection of bacteria in the gut ensures a varied repertoire of enzymes and metabolic pathways that contribute to health and homeostasis, and this state is referred to as eubiosis. Dysbiosis, on the other hand, refers to a disruption or change in the microbiota composition that may be associated with disease, and in the gut, this is reflected by a reduction in microbiota diversity. The gut microbiota has the ability to regulate circulating oestrogen levels via the estrobolome, which is defined as the collection of genes encoding oestrogen-metabolizing enzymes, specifically in the gut microbiome (Salliss *et al*., 2021).

An immense array of metabolic reactions occurs in the intestinal lumen, one being the deconjugation of oestrogen from its conjugate glucuronic acid (Salliss *et al*., 2021). This reaction requires the bacterial enzyme β-glucuronidase, found in specific gut bacteria such as, *Escherichia coli, Bacteroides fragilis* and *Streptococcus agalactiae* which can deconjugate glucuronide. The liver conjugates oestrogen, including 17β-oestradiol (E2) the predominant oestrogen in humans, with glucuronic acid (glucuronide) and secretes the glucuronide into bile salts, where the glucuronides are later released into the intestinal tract for excretion of the unused conjugated toxins and hormones (Baker *et al*., 2017). Glucuronide cannot be reabsorbed into the circulatory system. However, oestrogen that has been deconjugated from glucuronic acid, by the bacterial enzymatic action of β-glucuronidase, can be absorbed into the circulatory system as active oestrogen. Changes in the gut microbial composition, and thereby β-glucuronidase activity, could perturb or dysregulate circulating oestrogen levels and drive oestrogen-mediated conditions by contributing to hyperor hypo-estrogenic states (Salliss *et al*., 2021).

In the cervicovaginal microbiome*, Lactobacillus* dominance is associated with optimal gynaecologic and reproductive health. *Lactobacilli* create a competitive environment for invading pathogens and dysbiotic bacteria. Lactic acid, produced by *Lactobacillus*, lowers the vaginal pH to less than or equal to pH 4.5, and this low pH microenvironment is optimal for vaginal health. To survive and thrive, *Lactobacillus spp*. require glycogen by-products, which are provided by an oestrogen-dominant vaginal epithelium and host amylases (Spear *et al*., 2015). In addition, *Lactobacillus spp*. contribute to homeostasis by occupying this niche (pathogen exclusion) and by production of anti-inflammatory cytokines and antimicrobial peptides from epithelial cells, which fortifies the epithelial cell barrier (Spear *et al*., 2015).

## WHAT THEN IS THE ROLE OF THE LOCAL IMMUNE PROFILE?

Within the endometrial environment during this stage, known as ‘the implantation window’, a very peculiar influx of immune cells occurs and nearly completely switches local immunity from the inflammatory Th1 cell type to the tolerance-inducing Th2 cell type. This switch is crucial for implantation. During this period, 65-70% of the immune cells in the endometrium are uterine natural killer (uNK) cells that belong to the innate immunity compartment. Macrophages and dendritic cells are also detected, together with adaptive immune T cells, such as T regulatory cells (Tregs) (Mukherjee *et al*., 2023). Embryo attachment requires active local endometrial reactivity on the maternal side. The adhesion step is followed immediately by an anti-inflammatory reaction to enable the induction of the mechanisms of local tolerance, required for effective invasion. Early on, the ideal environment during the implantation window was thought to contain mainly Th2 (compared with Th1) cytokines, which would selectively allow the development of local mechanisms that promote immunotrophism and angiogenesis at the same time that they down-regulate inflammation and cytotoxic pathways (Marron & Harrity, 2019). Over time, the concept of pregnancy as a Th2 phenomenon has evolved: both the absence and a large excess of Th1 cytokines are thought to be deleterious for implantation and placentation, as is the absence of Th2 cytokines. This transient immune switch, together with the adequate uNK cell activation, appears fundamental in enabling the establishment of local maternal tolerance and survival of the foetus.

In endometriosis, the peritoneal environment is in a chronic state of local inflammation and contains immune cells with altered functions. This immune dysregulation in endometriosis creates an ideal environment for disease progression (Jiang *et al.,* 2021). At present, it is unclear whether immune dysfunction is a pathophysiological feature or a cause of endometriosis. In either case, there is a strong association demonstrated by the findings as reported by Jiang *et al.* (2021).

Many authors now believe that chronic endometriosis is in fact a disease of the local immune system in that the peritoneal macrophages of affected women display a decreased ability to phagocytosis but an up-regulated and increased activation of NF-_Ϗ_B pathways leading to the downstream upregulation of proinflammatory cytokines (TNF-α, IL-1β, and IL-6), proangiogenic factors (VEGF), growth factors and adhesion molecules (Fonseca *et al*., 2023).

T cell subset profiles are altered in women with endometriosis. There are higher numbers of Th17 cells in the peritoneal fluid of endometriosis patients, and consequently higher concentrations of IL-17 (Shi *et al*., 2022). The presence of elevated Th17 cells and IL-17 plays a major role in promoting chronic inflammation: IL-17 stimulates production of cytokines that induce angiogenesis and inflammation, contributing to the progression of endometriosis.

Females have a normal functioning immune system that competes with a semi allogenic conceptus during pregnancy. Thus, the acceptance and tolerance of the semi allogenic conceptus mandates the transformation of the maternal immune system. While the precise mechanisms by which the embryo is protected from the maternal immune assault are not fully understood, a picture that has emerged out of numerous studies suggests a dramatic transformation of multiple immune cells in the peripheral blood and endometrium. At the implantation stage, the immune cells, mainly the uterine natural killer (uNK) cells, T cells, dendritic cells, and macrophages make up half of the total number of endometrial cells. In order for a pregnancy to proceed, the role of the CD4+ CD25+ FOXP3+ regulatory T cells (T-regs) are important in mediating maternal immune tolerance to the allogeneic foetus during embryo implantation and early pregnancy. In general, pregnancy is associated with Th2 dominance, and Th1 immune response is associated with pregnancy losses. T helper 17 (Th17) cells differentiate from naive T-cells and produce the cytokine IL-17 that has an important role in the feto-maternal interface. During implantation, the Th17 cells are present in decidua and their numbers increase in the peripheral blood in the first trimester. However, it is proposed that lack of the T-regs (numerically as well as functionally) present in the endometrium leads to a subtle but constant inflammatory scenario which is not conductive to a successful implantation and live birth outcome. These immunosuppressive T cells work in conjunction with the uNKs which happen to be tolerance-inducing cells (unlike their peripheral blood counterparts which happen to be highly cytotoxic). In the endometrium, the uNKs are phenotypically CD3-CD56+, secreting cytokines such as TGF-β or IL10, thereby creating an immune supressed environment to allow implantation. It is an interesting but important fact that T-regs require the presence of commensals to become activated and when the microbiome is disturbed, the T-regs do not carry out their immune suppressive functions and control local inflammation.

## DOES THE ENDOMETRIAL MICROBIOME HAVE ANY ROLE TO PLAY?

The immediate answer is a resounding YES! There is abundant literature from IBS cases which clearly suggest that a state of dysbiosis with overgrowth by inflammatory bacteria leads to an immune profile conductive to chronic inflammation. It is believed that when the gut microbiome is disrupted and replaced by non-physiological commensals, the immune cells are typical of that of a dysregulated immune response with little to no tolerance to ingested foods, especially foreign proteins. The same applies to the endometrial surface: when the microbiome is replaced by endometrial pathogens (*Gardnerella, Ureoplasma, Enterococcus, Staphylococcus, E. coli*, and other mixed cultures), these bacteria induce an uncontrolled inflammatory response with all its consequences. To add to the injury, the predominant estrogenic milieu (due to lack of oestrogen-metabolizing bacteria) feeds the inflammatory cells and overtakes the tolerogenic cells (T-regs and uNKs) which are progesterone sensitive!

## CONCLUSIONS

It is logical to hypothesize that decreasing the numbers of bacterial pathogens in the reproductive tract and increasing the proportion of beneficial *Lactobacillus* could improve reproductive outcomes in those patients with an abnormal microbiota. The consequence of increasing the beneficial bacteria would ultimately lead to immune tolerance due to the presence of the Tregs and lower inflammatory cytokines. To address this, several approaches should be implemented: the use of antibiotics to eliminate the bacterial pathogens causing the dysbiosis, the use of probiotics specifically designed for reproductive health (high Lactobacilli probiotics) and hormonal balancing to control the raised oestrogen levels seen in these affected women. It is imperative to determine the immune profile of the cells in the endometrium (menstrual blood profiling) in order to monitor the beneficial changes induced by the therapy of choice and predict a possible positive outcome on the *in vitro* reproductive processes.
